# Phytochemical Composition, Health Benefits, Functional Properties, and Food Applications of Pumpkin Seeds

**DOI:** 10.1002/fsn3.71868

**Published:** 2026-05-08

**Authors:** Messenbet Geremew Kassa, Desye Alemu Teferi

**Affiliations:** ^1^ College of Agriculture, Food, Climate Science Injibara University Injibara Ethiopia

**Keywords:** bioactive compounds, food applications, functional foods, nutraceutical properties, pumpkin seeds

## Abstract

Pumpkin seeds ( *Cucurbita pepo* ) are increasingly recognized as a functional food due to their rich composition of proteins, healthy lipids, dietary fiber, and essential micronutrients. They have traditionally been consumed for culinary and therapeutic purposes. This review highlights the chemical composition, bioactive compounds, health‐promoting potential, and applications of pumpkin seeds in food systems. Pumpkin seeds provide high‐quality proteins, unsaturated fatty acids, and minerals such as magnesium, zinc, iron, and potassium, supporting cardiovascular health, immune function, and gastrointestinal well‐being. They also contain phytosterols, antioxidants (vitamin E, carotenoids), cucurbitacins, lignans, and tryptophan, which have been associated with cholesterol reduction, oxidative stress mitigation, anti‐inflammatory effects, hormonal regulation, and neurochemical benefits in preclinical studies. From a food science perspective, pumpkin seeds are versatile, being incorporated into snacks, bakery products, salads, soups, functional foods, and dietary supplements. Pumpkin seed oil is valued for its favorable fatty acid profile and antioxidant content. Integration of pumpkin seeds into food formulations enhances both nutritional quality and functional properties. Continued research is warranted to explore their bioactivity, health effects in humans, and innovative applications in modern diets.

## Graphical abstract

Pumpkin seeds (*Cucurbita* spp.) are rich in bioactive compounds including proteins, unsaturated fatty acids, dietary fiber, vitamins (E and B‐complex), minerals (zinc, magnesium, iron), and phytochemicals such as phenolics, flavonoids, and phytosterols. These components contribute to various health benefits, including antioxidant, anti‐inflammatory, cardioprotective, antidiabetic, and anticancer effects. Functionally, pumpkin seed components exhibit desirable properties such as emulsification, water and oil absorption, and foaming capacity, making them suitable for food formulation. The seeds and their derivatives (flour, oil, protein isolates) have wide applications in bakery, snack products, functional foods, and nutraceuticals. Overall, pumpkin seeds represent a sustainable and nutrient‐dense ingredient with significant potential in promoting human health and enhancing food quality.

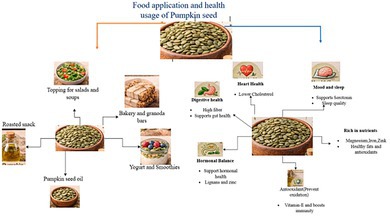

## 1 Introduction

Pumpkin seeds, also known as pepitas, have gained significant recognition for their impressive nutritional profile and diverse health benefits (Wal et al. 2024). Pumpkin ( *Cucurbita pepo* ) seeds have long been used as both a food ingredient and a source of therapeutic benefits (Golija 2019). The unique chemistry, nutraceutical potential, and versatile food applications of pumpkin seeds highlight their growing importance in modern nutrition and health (Hadidi et al. 2025). Beyond fats and proteins, pumpkin seeds are rich in dietary fiber, promoting regular bowel movements and supporting a healthy gut microbiome (Kumari et al. 2025). The seeds are also packed with vitamins and minerals, including magnesium, zinc, iron, and potassium (Syed et al. 2019). Magnesium plays a crucial role in maintaining cardiovascular health, bone density, and muscle function, while zinc is essential for immune function and wound healing. Iron is vital for oxygen transport in the blood, and potassium helps regulate fluid balance and blood pressure (Haddy et al. 2006).

The nutraceutical potential of pumpkin seeds is attributed to their high content of various bioactive compounds (Lestari and Meiyanto 2018). Notably, pumpkin seeds contain phytosterols, plant‐derived compounds that help lower cholesterol levels and reduce the risk of heart disease. Pumpkin seeds are also rich in antioxidants, including vitamin E and carotenoids, which protect cells from oxidative damage and inflammation, supporting overall health and disease prevention (Syed et al. 2019). Another significant bioactive component is cucurbitacin, a type of triterpenoid that has been studied for its potential anti‐cancer properties (Zieniuk and Pawełkowicz 2023). Cucurbitacin exhibits inhibitory effects on cancer cell proliferation and may contribute to the prevention and management of various cancers. Furthermore, pumpkin seeds are rich in lignans, which have been associated with reduced risk of certain cancers and improved hormonal balance (Shruti et al. 2024). The seeds also contain amino acids such as tryptophan, which is a precursor to serotonin. Serotonin plays a key role in regulating mood, sleep, and appetite, suggesting that pumpkin seeds may have potential benefits in managing mood disorders and improving sleep quality (Wal et al. 2024).

The versatility of pumpkin seeds makes them an excellent ingredient in a variety of food applications (Hussain, Kausar, Sehar, et al. 2022a, 2022b; Hussain, Kausar, Murtaza, et al. 2022). Their mild, nutty flavor and crunchy texture enhance both sweet and savory dishes. In culinary practices, pumpkin seeds are used as a topping for salads, soups, and yogurt, or incorporated into baked goods such as bread, muffins, and granola bars (Bashir et al. 2025). They are also roasted and salted to create a popular snack. In addition to their use in traditional food products, pumpkin seeds are increasingly being utilized in functional foods and dietary supplements (Hussain, Kausar, Sehar, et al. 2022a, 2022b; Hussain, Kausar, Murtaza, et al. 2022). They are often included in formulations designed to support cardiovascular health, enhance digestive function, and improve overall wellness. Pumpkin seed oil, extracted from the seeds, is used in salad dressings, marinades, and as a finishing oil for various dishes (Šamec et al. 2022). The oil is valued for its high content of unsaturated fatty acids and antioxidants, making it a healthful addition to the diet. The incorporation of pumpkin seeds into various food products not only enhances nutritional value but also provides potential health benefits (Devi et al. 2018). Their diverse applications highlight their importance in modern diets and underscore the ongoing research into their functional properties.

## 2 Methodology

**FIGURE 1:**
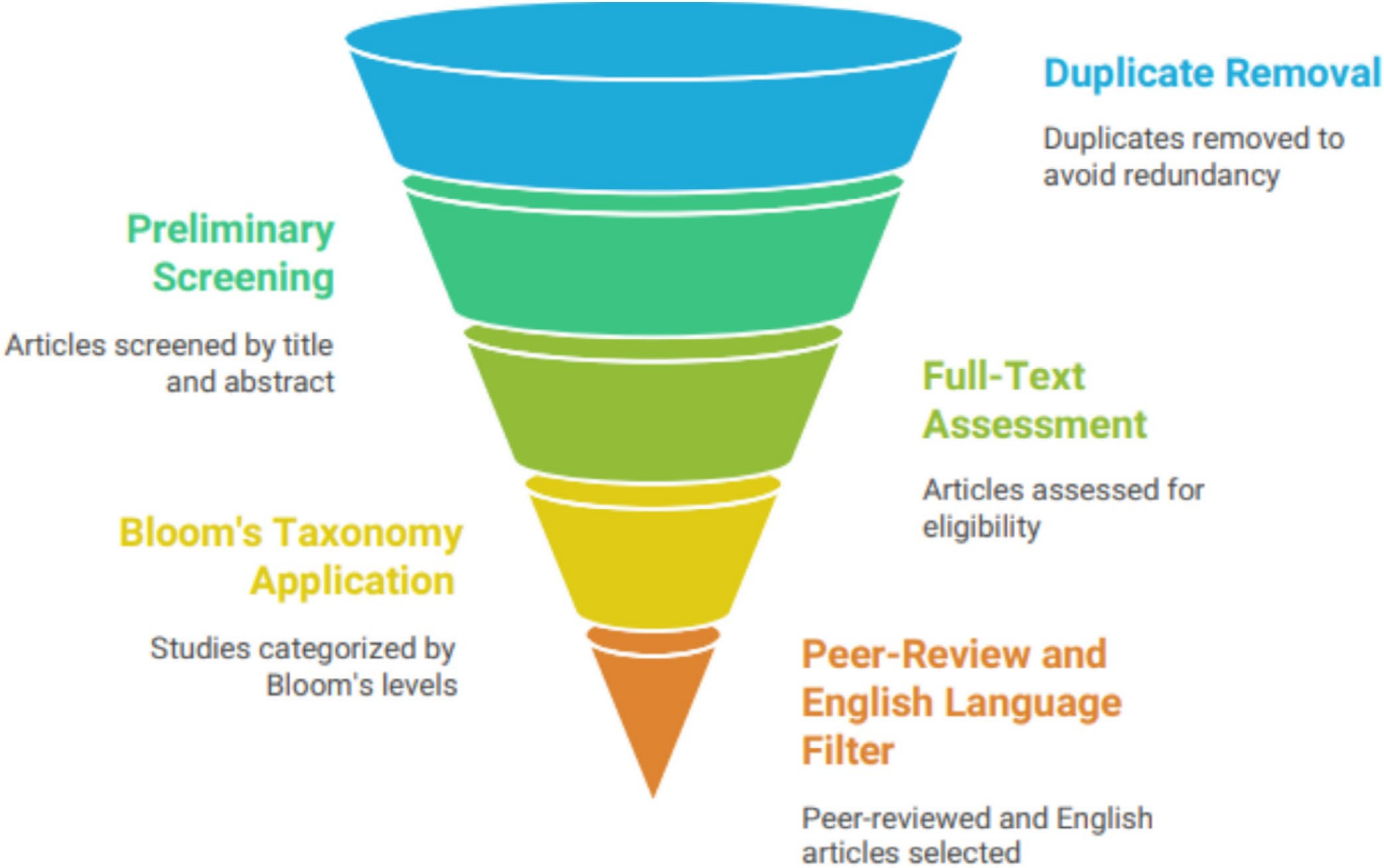
Pumpkin seed literature review process.

This review adopted a systematic approach to collect, evaluate, and synthesize scientific information on the phytochemical composition, health benefits, functional properties, and food applications of pumpkin seeds ( *Cucurbita pepo* ) (Figure 1). Relevant literature was retrieved from major scientific databases, including Scopus, Web of Science, PubMed, ScienceDirect, and Google benefits” OR “antioxidant activity” AND “functional properties” OR “food applications.” Scholar, covering publications up to 2026. A comprehensive search strategy was applied using Boolean operators (“AND” and “OR”) to combine keywords such as “pumpkin seeds” OR “ *Cucurbita pepo* ” AND “phytochemical composition” OR “bioactive compounds” AND “health.

A total of 650 articles were initially identified through database searching. After removing duplicates and conducting preliminary screening, 500 studies were retained based on title and abstract relevance. Subsequently, 460 articles underwent full‐text assessment according to predefined eligibility criteria, resulting in the final inclusion of 106 studies for comprehensive analysis (Table 1).

**TABLE 1:** Summary of literature screening and inclusion process.

Stages	Description	Number of articles
Initial database search	Records identified from Scopus, Web of Science, Science Direct, PubMed, and Google Scholar	650
Duplicate removal and abstract screening	Excluded non‐ relevant and duplicate records	500
Full‐ text assessment	Articles were evaluated for detailed eligibility	460
Final inclusion	Studies meeting all inclusion criteria	106

The study selection process was guided by principles adapted from Bloom's Taxonomy, enabling structured coverage of the literature. Foundational studies describing chemical composition and nutritional value were considered at the understanding level, while studies addressing functional properties and food applications were included at the application level. Mechanistic and comparative studies on antioxidant, anti‐inflammatory, and disease‐preventive effects were evaluated at the analysis level, and high‐quality reviews and critical studies were emphasized at the evaluation level.

Only peer‐reviewed articles, review papers, and book chapters published in English were included, whereas duplicate records, non‐scientific reports, and irrelevant studies were excluded. Articles were screened based on titles, abstracts, and full texts to ensure relevance and quality.

Key data extracted from the selected studies included phytochemical constituents (such as phytosterols, tocopherols, carotenoids, and phenolic compounds), nutritional composition (proteins, lipids, and dietary fiber), functional properties (including emulsification, foaming, and water absorption), documented health benefits, and applications in food systems. The collected information was critically analyzed and organized into thematic sections, with emphasis on recent findings and scientifically validated results to ensure the reliability and comprehensiveness of the review.

## 3 Chemistry of Pumpkin Seeds

Pumpkin seeds, scientifically known as pepitas, are small, green seeds encased in a white hull (Adsul and Madkaikar 2020). They are harvested from the fruit of the pumpkin plant ( *Cucurbita pepo* ) and have long been valued for their dense nutritional content and potential health benefits. The chemistry of pumpkin seeds is complex, involving a wide array of macronutrients, micronutrients, and bioactive compounds that contribute to their status as a superfood.

### 3.1 Macronutrients

#### 3.1.1 Proteins

Pumpkin seeds are an excellent source of plant‐based protein, containing approximately 30%–39% protein by weight (Hadidi et al. 2025). The protein profile of pumpkin seeds includes all essential amino acids, making them a complete protein source. Notably, they are rich in glutamic acid, arginine, and tryptophan. Glutamic acid is vital for brain function, arginine is known for its role in cardiovascular health, and tryptophan is a precursor to serotonin, a neurotransmitter that regulates mood and sleep.

Amino acids play crucial roles both as building blocks of proteins and as intermediates in metabolism. The dietary intake of sufficient quantities and high‐quality essential amino acids is vital for physiological functions in the human body. Studies indicate that protein isolates from pumpkin seeds are similar to those of soybeans, exhibiting high bioavailability of amino acids (Habib et al. 2025). Zhao et al. (2025) have elucidated that the globulin structure of pumpkin seed protein is analogous to that of legume seeds. This nutritional similarity is significant because it supports the use of pumpkin seed protein as a reliable ingredient in the formulation of nutritious food recipes, thereby helping to mitigate the effects of protein malnutrition in vulnerable communities. Additionally, protein isolates from pumpkin seeds possess promising antioxidative and chelating properties (Vinayashree and Vasu 2021).

##### 3.1.1.1 Functional Properties of Pumpkin Seed Protein

###### 3.1.1.1.1 Foaming Properties

Foaming capacity (FC) and foaming stability (FS) are critical indicators used to assess the techno‐functional properties of proteins for diverse food applications. Proteins such as pumpkin seed protein (PSP) have demonstrated strong foaming abilities, even at low concentrations, due to their capacity to rapidly encapsulate air bubbles, a function influenced by molecular flexibility, net surface charge, and hydrophobic interactions. Traditional foaming agents like egg white and soy proteins are widely utilized in the food industry for their superior foaming performance (Sá et al. 2023).

The functional performance of PSP can be significantly modulated through various processing techniques. Among these, thermal processing, microwave irradiation, and ultrasonic treatments have all been shown to enhance foaming properties, with conventional thermal methods producing the most pronounced improvements in FC. The addition of salts further contributes to increased FS by promoting protein solubility and interfacial activity (Sá et al. 2023). Notably, high‐intensity ultrasound treatment has been identified as particularly effective in enhancing both FC and FS of PSP. Studies by (Du et al. 2022; Sert et al. 2022) demonstrated that such treatment significantly improved PSP's foaming behavior across a range of pH values. Post‐treatment, FC values were observed to nearly double compared to untreated samples, indicating a marked enhancement in the protein's foaming potential.

###### 3.1.1.1.2 Emulsifying Properties

The emulsifying properties of proteins play a pivotal role in a wide range of food applications, including cake batters, coffee whiteners, chopped and comminuted meats, mayonnaise, salad dressings, and frozen desserts (Hadidi et al. 2025). These properties are typically characterized by two parameters: emulsion activity (EA), which reflects a protein's ability to form emulsions, and emulsion stability (ES), which indicates the capacity of the emulsified droplets to remain uniformly dispersed without undergoing creaming, coalescence, or flocculation. Several intrinsic and extrinsic factors such as molecular weight, solubility, net charge, hydrophobicity, temperature, ionic strength, pH, and conformational stability profoundly influence these attributes (Vinayashree and Vasu 2021).

Pumpkin seed protein (PSP) has emerged as a promising emulsifying agent, exhibiting notably high EA. Research conducted by (Vinayashree and Vasu 2021) revealed significant variation in emulsifying performance among different PSP fractions. Among these, the water‐soluble fraction demonstrated the greatest emulsion stability, underscoring its potential utility in formulations where long‐term emulsion integrity is critical.

###### 3.1.1.1.3 Water‐Binding Capacity

The water‐binding capacity of pumpkin seed protein is a fundamental parameter influencing several key techno‐functional properties relevant to food processing and product formulation (Hadidi et al. 2025). Among these, water‐binding capacity (WBC) also referred to as water‐holding capacity (WHC) is particularly critical, as it denotes the ability of proteins to retain water against gravitational forces. This property plays a vital role in determining the texture, moisture retention, and shelf life of various food products (Jaenicke 1991). WBC is intricately linked to the amino acid composition and the three‐dimensional structural conformation of protein molecules. A higher prevalence of charged amino acids often correlates with increased water retention. Moreover, several factors such as molecular structure, hydrophobicity, pH, temperature, protein concentration, and ionic strength can significantly affect WBC (Mapiour and Amira 2023). Despite its potential, pumpkin seed protein (PSP) preparations often display relatively low water‐binding capacities (Mapiour and Amira 2023). This limitation is possibly attributed to their high lipid content and a limited presence of non‐polar amino acids such as valine, tyrosine, glycine, and proline (Mapiour and Amira 2023).

In a study conducted by (Hadidi et al. 2025), PSP concentrates were prepared using different aqueous extraction methods, including salt extraction (SE) and alkaline extraction–isoelectric precipitation (AE‐IP). The results demonstrated notable variations in both WBC and oil‐binding capacity (OBC) based on the extraction technique. AE‐IP samples generally exhibited superior WBC compared to SE samples, likely due to higher protein content and stronger protein‐water interactions. Conversely, acetylation of PSP significantly enhanced both WBC and oil absorption capacity (OAC). Acetylated protein concentrates exhibited improved fluid‐binding properties due to the incorporation of hydrophilic and lipophilic functional groups during modification. These enhanced functional traits make them particularly suitable for incorporation into viscous food matrices and as extenders in processed meat products (Hadidi et al. 2025).

Moreover, PSP has shown potential for zinc fortification, as demonstrated by (Peng et al. 2022). Their study assessed the zinc‐binding capacity of various PSP hydrolysates (PSPHs), quantifying it as the percentage of zinc bound relative to the total zinc introduced. Binding capacities ranged from 39.3% to 76.3%, with the most effective PSPH containing the highest concentration of amino acids, suggesting that peptide composition and chain length are decisive factors in mineral chelation efficiency. Overall, the binding capacities of pumpkin seed protein, whether for water, oil, or trace minerals, are central to enhancing its functional applications in food systems (Pandey et al. 2024). Continued research into the molecular interactions governing protein‐fluid relationships will be critical for optimizing PSP utilization in the development of stable, texturally desirable, and nutritionally enhanced food products.

###### 3.1.1.1.4 Gelation Properties

Heat‐induced gelation is a fundamental process in food formulation, critical for achieving desirable gel textures that enhance flavor release and overall sensory quality. Upon thermal treatment, protein molecules undergo structural unfolding, facilitating the formation of intermolecular aggregates through hydrophobic interactions, hydrogen bonding, and electrostatic forces (Rajan et al. 2021). These interactions lead to the development of a cohesive three‐dimensional gel matrix. However, plant‐based proteins often form weaker gels upon heat induction, thereby limiting their applicability in various food systems.

To address these limitations, a range of pretreatment strategies including physical, chemical, and enzymatic methods have been explored to improve the gel‐forming ability of plant proteins. Notably, (Hadidi et al. 2025) investigated the partial substitution of egg‐white protein with pumpkin seed protein (PSP). Their findings indicated that increasing the proportion of PSP in the protein matrix led to diminished gel strength. Nonetheless, the microstructural analysis of these mixed gels revealed distinct domains of PSP aggregates embedded within the egg‐white protein network (Tomczyńska‐Mleko et al. 2023). This heterogeneity produced a visibly rougher gel surface compared to gels composed solely of egg‐white protein. Overall, the gelation behavior of pumpkin seed protein is highly dependent on pretreatment techniques such as ultrasonication and pH‐shifting, which have demonstrated potential to enhance gel structure and performance (Zeng et al. 2023). A deeper understanding of these modulatory factors is essential for optimizing the functional properties of PSP and broadening the applicability of plant proteins in advanced food product development.

#### 3.1.2 Fats

Pumpkin seeds are also high in healthy fats, with a lipid content of about 45%–50% (Wal et al. 2024). These fats are predominantly unsaturated, including omega‐6 fatty acids (linoleic acid) and monounsaturated fatty acids (oleic acid) (Odabaşioğlu 2024). Linoleic acid is essential for maintaining skin health and metabolic functions, while oleic acid is known for its heart‐healthy properties. Additionally, pumpkin seeds contain a small amount of omega‐3 fatty acids, which have anti‐inflammatory effects and contribute to heart health (Wal et al. 2024). The principal fatty acids in pumpkin seed oil (PSO) are linoleic, oleic, stearic, and palmitic acids, which together constitute more than 95% of the total fatty acids, with about 75% being unsaturated fatty acids (UFAs) (Šamec et al. 2022). Additionally, small concentrations of arachidic and linolenic acids have also been reported. The fatty acid profile of PSO is detailed in Table 2. Unsaturated fatty acids have been extensively studied due to their protective effects against cardiovascular diseases (Sokoła‐Wysoczańska et al. 2018). They are crucial for the healthy growth and development of the brain and nervous system and are known to have health benefits in ameliorating coronary heart disease, hypertension, and arthritis, as well as in reducing inflammation, autoimmune‐related disorders, and cancer. Moreover, linoleic and alpha‐linolenic acids are the only two fatty acids considered essential for humans, as they cannot be synthesized by the human body and must be obtained through diet (Tang et al. 2018).

**TABLE 2:** Nutritional composition of pumpkin seeds (dry base).

Components	Content (mg/100 g)	References
Crude protein (mg)	14.05–39.25	(Mohammed 2004; Polyzos et al. 2024)
Crude fat (mg)	31.90–58.0	(Mohammed 2004; Polyzos et al. 2024)
Total ash (mg)	3.52–4.59	(Mohammed 2004; Polyzos et al. 2024)
Crude fiber (mg)	16.84	(Mohammed 2004)
Carbohydrates (mg)	11.69–24.85	(Mohammed 2004; Polyzos et al. 2024)
Total fiber (mg)	46.65	(Dotto and Chacha 2020)
Sodium (mg)	1.35	(Dotto and Chacha 2020)
Potassium (mg)	434.71	(Dotto and Chacha 2020)
Iron (mg)	6.02	(Dotto and Chacha 2020)
Calcium (mg)	4.00	(Dotto and Chacha 2020)
Zink (mg)	18.78	(Dotto and Chacha 2020)
Phosphorus (mg)	0.74	(Dotto and Chacha 2020)
Copper (mg)		(Dotto and Chacha 2020)

#### 3.1.3 Carbohydrates and Fiber

While pumpkin seeds are low in overall carbohydrates (10%–16.84%), they are an excellent source of dietary fiber, particularly insoluble fiber (Li, Wu, et al. 2022; Li, Xin, et al. 2022). This fiber aids in digestive health by promoting regular bowel movements and supporting a healthy gut microbiome (Slavin 2013). The fiber content also contributes to a feeling of fullness, which can be beneficial for weight management.

### 3.2 Micronutrients

#### 3.2.1 Vitamins

Pumpkin seeds are packed with essential vitamins (Table 3), including vitamin E (as gamma‐tocopherol); B‐vitamins such as riboflavin, niacin, and pantothenic acid; and vitamin K (Sánchez‐Velázquez et al. 2023). Vitamin E acts as a powerful antioxidant, protecting cells from oxidative damage. B‐vitamins play crucial roles in energy metabolism, red blood cell formation, and neurological function. Vitamin K is essential for blood clotting and bone health (Maqbool et al. 2017). Pumpkin seeds are also a good source of vitamin E, which includes four tocopherol and tocotrienol isomers (α, β, γ, and δ) (Butinar et al. 2011). These isomers differ in the number and position of the methyl groups on the chromanol ring. However, only one isomer, d‐RRR‐α‐tocopherol, meets the criteria of being a true vitamin E (Zhao et al. 2015). While sunlight can effectively trigger the synthesis of vitamin E in the human body, many plant species, including pumpkin seeds, are rich sources of this vitamin. Pumpkin seeds are particularly rich in tocopherols, with γ‐tocopherol being the dominant isomer, followed by α‐ and δ‐tocopherols (Šamec et al. 2022). They also contain small amounts of α‐tocotrienol, β‐ and γ‐tocotrienols. These tocopherols and tocotrienols are powerful antioxidants capable of deactivating highly reactive radicals by releasing H^+^ ions from their rings (Budzianowska et al. 2025). This process helps protect cell lipids from peroxidation, thereby reducing the risk of oxidative damage.

**TABLE 3:** Vitamin content of pumpkin seeds.

Vitamins in pumpkin seed	Amount	References
Folates	0.058 mg	(Kwiri et al. 2014; Devi et al. 2018)
Niacin	4.99 mg	(Kwiri et al. 2014; Devi et al. 2018)
Pantothenic acid	0.750 mg	(Kwiri et al. 2014; Devi et al. 2018)
Pyridoxine	0.143 mg	(Kwiri et al. 2014; Devi et al. 2018)
Riboflavin	0.153 mg	(Kwiri et al. 2014; Devi et al. 2018)
Thiamin	0.273 mg	(Kwiri et al. 2014; Devi et al. 2018)
Vitamin A	16 IU	(Kwiri et al. 2014; Devi et al. 2018)
Vitamin C	0.0019 mg	(Devi et al. 2018)
Vitamin E	35.10 mg	(Devi et al. 2018)

Tocopherols may also act as prooxidants, reducing the quantity of transition metals in tissues, although this mechanism is highly dependent on the tocopherol levels (Ansari et al. 2025). While the primary role of tocopherols in pumpkin seed is as antioxidants, they also have non‐antioxidant functions. Recently, γ‐tocopherol has been found effective in neutralizing peroxynitrite, a potent oxidant with a wide range of harmful effects on cells (Es‐Sai et al. 2025). This has increased interest in the cell signaling‐related activities of tocopherols. Studies have shown that tocotrienols in pumpkin seed possess considerable anticholesterolemic potential, a property unique to tocotrienols, as well as neuroprotective, cardioprotective, and antitumor properties (Wal et al. 2024). Tocotrienols share several functional features with isolated tocopherols in vitro and are measurable in plasma in both human and animal subjects. However, there is limited information on their bioaccumulation in human tissues.

#### 3.2.2 Minerals

Minerals are abundant in pumpkin seeds, with significant levels of magnesium, zinc, iron, copper, and manganese (Ahmad et al. 2025). Magnesium is critical for muscle and nerve function, bone health, and energy production. Zinc is essential for immune function, protein synthesis, and DNA repair. Iron is vital for oxygen transport in the blood, while copper and manganese are important for various enzymatic reactions in the body. Pumpkin seeds contain a significant amount of valuable minerals. They are rich in potassium (K) and relatively low in sodium (Na), and they have high levels of calcium (Ca), manganese (Mn), phosphorus (P), and magnesium (Mg) (Singh and Kumar 2022). Pumpkin seeds are also a good source of trace elements such as zinc (Zn), iron (Fe), and copper (Cu) (Yetesha et al. 2023). Minerals like Zn, Cu, Mn, and Fe possess antioxidant properties and serve as cofactors for vital antioxidant‐dependent enzymes. The low sodium and high potassium content in pumpkin seeds have important clinical implications for improving cardiovascular health (Adnan et al. 2025). Zinc is essential for male reproduction, structural proteins, and cellular protection. These mineral concentrations make pumpkin seeds a valuable ingredient for food fortification, particularly in bakery products.

### 3.3 Bioactive Compounds

#### 3.3.1 Phytosterols

Pumpkin seeds are rich in phytosterols, primarily beta‐sitosterol, which have been shown to lower cholesterol levels and improve heart health (Arora et al. 2023). Phytosterols compete with dietary cholesterol for absorption in the intestines, reducing overall cholesterol uptake. Pumpkin seeds are a rich source of phytosterols, a group of naturally occurring compounds structurally similar to cholesterol (Wal et al. 2024). These bioactive compounds are primarily recognized for their cholesterol‐lowering properties. The most prevalent phytosterols in pumpkin seeds include beta‐sitosterol, stigmasterol, and campesterol. Phytosterols exert their cholesterol‐lowering effects by competing with dietary cholesterol for absorption in the intestines (Li, Wu, et al. 2022; Li, Xin, et al. 2022). This competition reduces the amount of cholesterol absorbed into the bloodstream, thereby lowering overall blood cholesterol levels. This mechanism is particularly beneficial in reducing LDL (“bad”) cholesterol, which is a significant risk factor for cardiovascular diseases. In addition to their cholesterol‐lowering effects, phytosterols possess anti‐inflammatory and antioxidant properties. They help reduce oxidative stress by neutralizing free radicals, thereby protecting cells from damage. This antioxidative capacity contributes to the prevention of chronic diseases, including heart disease and cancer. Moreover, phytosterols have been shown to enhance immune function and reduce inflammation, which are critical in the prevention and management of various inflammatory conditions (Nattagh‐Eshtivani et al. 2022).

#### 3.3.2 Cucurbitacins

Cucurbitacins are triterpenoid compounds found in pumpkin seeds, known for their bitter taste and potent pharmacological effects, as detailed in Table 4. Cucurbitacins are a group of highly bioactive triterpenoid compounds found in pumpkin seeds (Wal et al. 2024). These compounds are known for their distinctive bitter taste and potent pharmacological properties. The primary types of cucurbitacins identified in pumpkin seeds include cucurbitacin A, B, C, D, E, and I (Lin et al. 2024). Each type of cucurbitacin has unique structural variations that contribute to its specific biological activities. They exhibit significant anticancer properties by inhibiting cancer cell proliferation, inducing apoptosis, and preventing metastasis. Cucurbitacins also have anti‐inflammatory effects, reducing the activity of pro‐inflammatory enzymes and cytokines, and possess antioxidant activity, which helps neutralize free radicals and protect against oxidative stress (Silvestre et al. 2022).

**TABLE 4:** Phytochemicals in Pumpkin Seeds and their extraction methods.

Phytochemicals	Extraction methods	References
Fatty acids like palmitic, stearic, oleic and linoleic acids, sulfur‐containing amino acids, and phytosterols	Chromatography	(Ahmad and Khan 2019)
Tocopherols, squalene, and sterols	Spray‐drier	(Ogrodowska et al. 2017)
Phenolic content and antioxidants	HPLC and U.V. Spectrophotometer	(Saavedra et al. 2015)
Carotenoids	Spectrophotometer	(Wongsagonsup et al. 2015)
Tocopherols, sterols, β‐carotene, and lutein	Cold pressing and solvent extraction methods	(Stajčić et al. 2022)

## 4 Health Benefits

### 4.1 Anti‐Diabetic Effect

Pumpkin seeds are rich in phenolic antioxidant compounds, including trigonelline, D‐chiro‐inositol, and nicotinic acid, which are recognized as insulin action mediators or insulin sensitizers (Dowidar et al. 2020). These bioactive constituents exert multiple physiological effects, particularly on pancreatic β‐cell function, insulin secretion, and the regulation of key enzymes involved in glucose metabolism (Adams et al. 2014). D‐chiro‐inositol, one of the principal components, is believed to function as an intracellular mediator of insulin signaling (Gambioli et al. 2021). It facilitates glycogen synthase dephosphorylation, activates pyruvate dehydrogenase, and enhances the activity of rate‐limiting enzymes in both oxidative and non‐oxidative glucose uptake pathways (Larner 2002). Trigonelline, a bioactive plant alkaloid found in pumpkin seeds, has demonstrated significant potential in alleviating hyperglycemia and hyperlipidemia. Prior research by (Yoshinari et al. 2009) indicated that both trigonelline and nicotinic acid enhance the activity of critical enzymes in glucose metabolism, including glucokinase and glucose‐6‐phosphatase. Additionally, another study by (Caili et al. 2006) highlighted the presence of a diverse range of protein constituents in pumpkin seeds, with molecular weights ranging from 3 to 60 kDa, which may contribute to improved insulin levels and enhanced glucose tolerance.

### 4.2 Anticancer Properties

**FIGURE 2:**
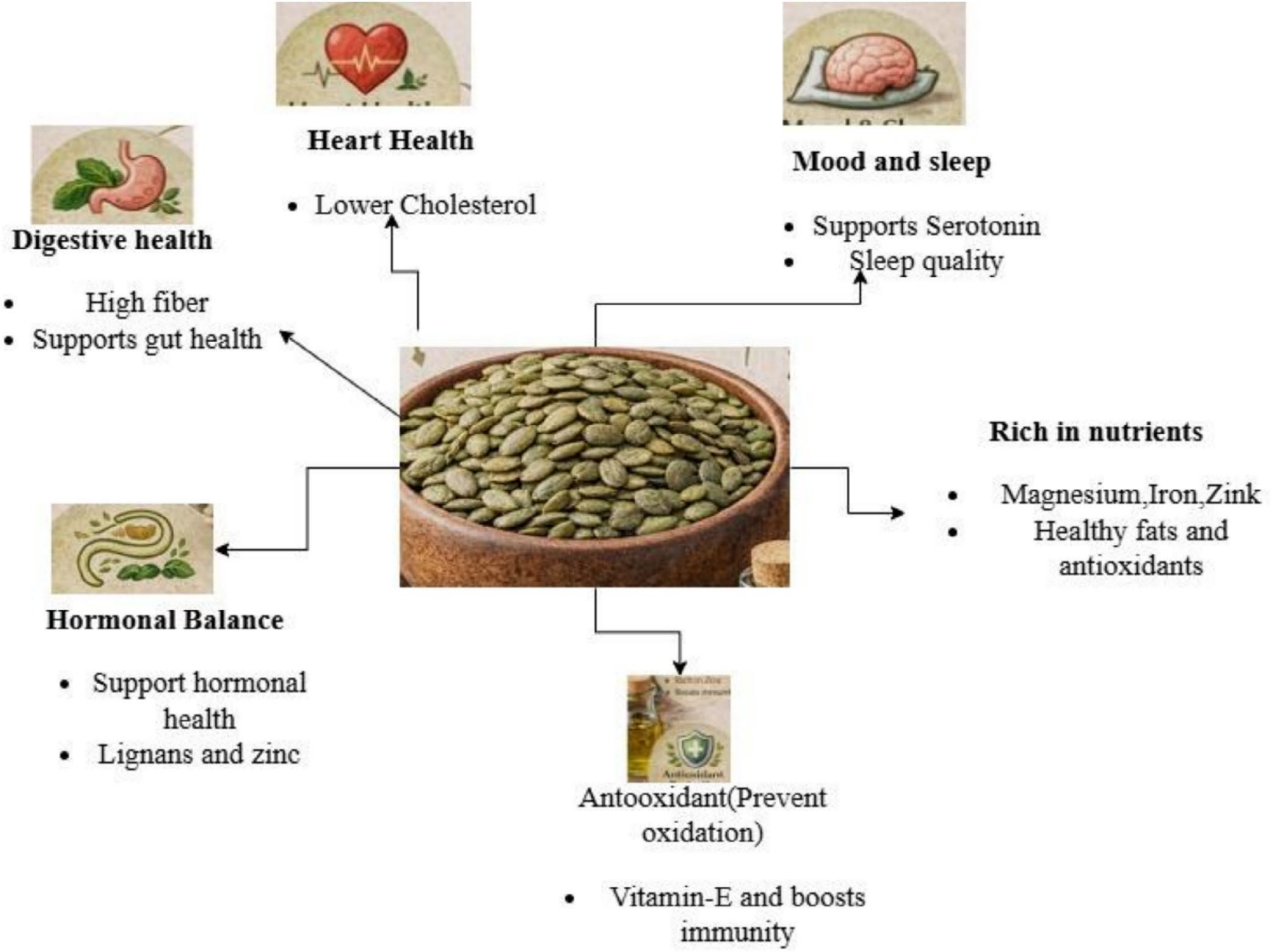
Health benefits of pumpkin seeds.

Phytoestrogens exhibit a controversial influence on hormone‐dependent tumors (Domínguez‐López et al. 2020). Previous studies using a breast cancer rat model have demonstrated that treatment with pumpkin seed extract can modulate hormonal activity (Figure 2) by enhancing estradiol production and altering the expression of estrogen receptor (ER)‐α, ER‐β, and progesterone receptor (PR) in MCF7, BeWo, and Jeg3 breast cancer cell lines. In this context, (Richter et al. 2010) observed a dose‐dependent increase in estradiol synthesis across all three cell types. Notably, in MCF7 cells, a significant downregulation of ER‐α expression accompanied by an upregulation of PR was reported. These findings suggest that pumpkin seed compounds may hold therapeutic potential in the treatment and/or prevention of hormone‐dependent breast cancers through modulation of estrogen and progesterone signaling pathways.

(Chari et al. 2018) conducted an in vivo study to evaluate the effect of ethanolic pumpkin seed extract on 1,2‐dimethylhydrazine‐induced colon cancer in Wistar rats. Their findings showed that administration of 200 mg/kg of the extract induced apoptosis in cancer cells, indicating its cytotoxic potential against tumor tissues. Similarly, other studies have reported that hydroalcoholic extracts of pumpkin seeds can inhibit the proliferation of human hepatocarcinoma (HepG2) and colon carcinoma (CT26) cell lines (Kalita et al. 2024). In addition, (Al‐Sharqawi et al. 2024) demonstrated antiproliferative activity of pumpkin seed extracts in breast, colon, and lung cancer cell lines. (Nomikos et al. 2021) further reported a dose‐dependent inhibitory effect of cucurbitacin, a bioactive compound isolated from pumpkin seeds, on prostate cancer cell lines. Their study also revealed that cucurbitacin induces cell cycle arrest, disrupts cytokinesis, and enhances apoptosis.

While several researchers attribute the anticancer potential of pumpkin seeds to their flavonoid and cucurbitacin content (Wal et al. 2024; Zieniuk and Pawełkowicz 2023), others suggest that cucurbitacin alone may not fully explain these effects (Chari et al. 2018). This discrepancy indicates that additional bioactive compounds or mechanisms may contribute to the observed anticarcinogenic activity. Therefore, further studies focusing on compound isolation, mechanistic pathways, and advanced analytical approaches are necessary to better understand and validate these effects.

### 4.3 Antimicrobial Effects

**FIGURE 3:**
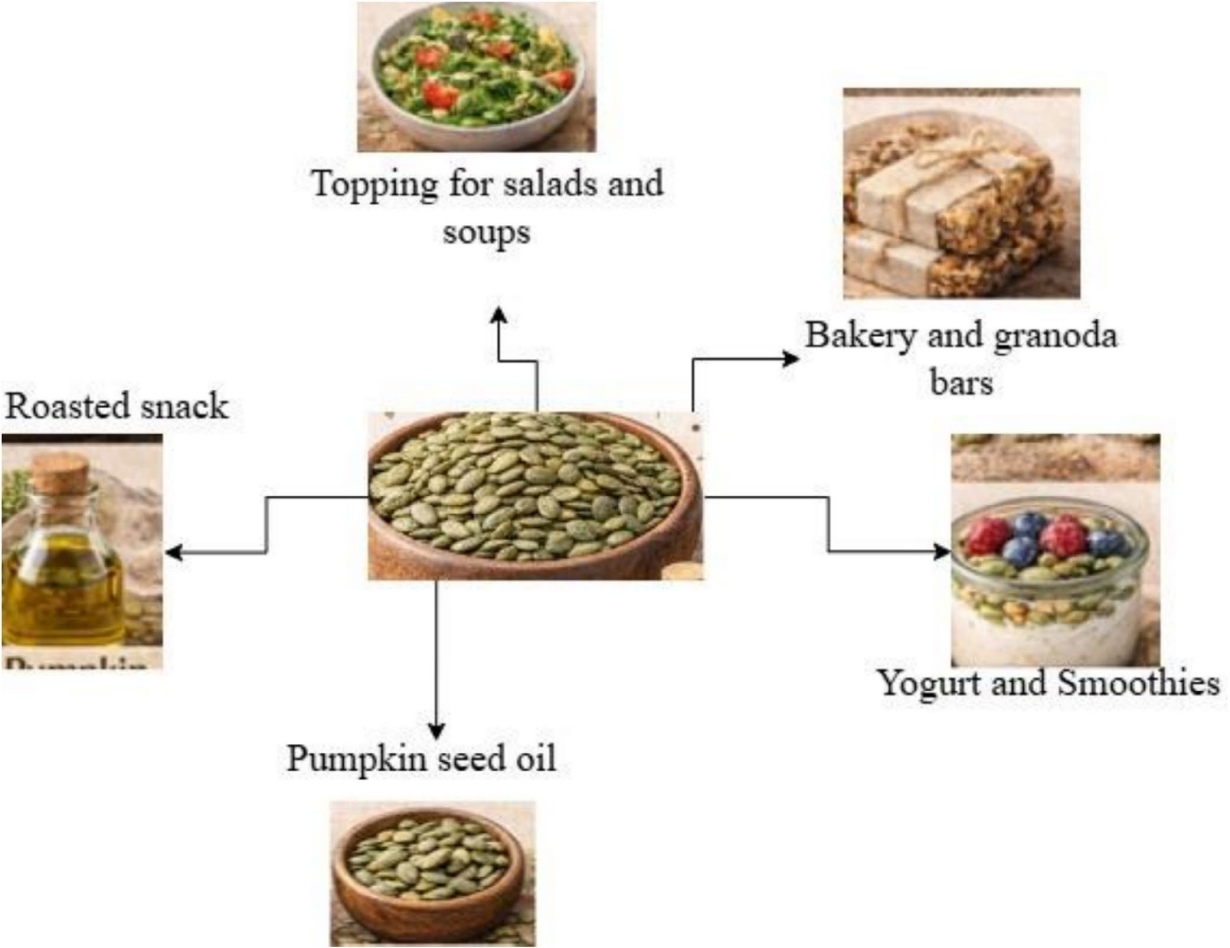
Applications of pumpkin seed in food product development.

Pumpkin seeds possess notable antimicrobial properties attributed to their rich content of bioactive compounds such as phenolics (Figure 3), flavonoids, and essential fatty acids (Hussain, Kausar, Sehar, et al. 2022a, 2022b; Hussain, Kausar, Murtaza, et al. 2022). These compounds have demonstrated inhibitory effects against a range of pathogenic microorganisms, including bacteria and fungi. Studies have shown that pumpkin seed extracts can disrupt microbial cell membranes, interfere with enzyme activity, and inhibit microbial growth (Petropoulos et al. 2021). Specifically, the presence of compounds like cucurbitacins and certain peptides has been linked to strong antibacterial activity, particularly against Gram‐positive and Gram‐negative bacteria (Nagarajappa et al. 2024). These antimicrobial attributes support the potential application of pumpkin seeds as a natural preservative or therapeutic agent in food and pharmaceutical industries.

A 28‐kDa antifungal protein classified as PR‐5 (pathogenesis‐related protein 5), isolated from pumpkin seeds, has been shown to significantly inhibit the growth of *Fusarium oxysporum* on agar plates at concentrations exceeding 2 μM (Yadav et al. 2013). This protein also exhibited synergistic antifungal activity when combined with nikkomycin, a chitin synthase inhibitor, effectively suppressing the growth of *Candida albicans* (Dowidar et al. 2020). In a separate study, (Park et al. 2010) identified another antifungal protein, approximately 40 kDa in molecular weight, from the pumpkin rind, which they designated as PR‐1. This protein demonstrated strong antifungal activity against *Fusarium oxysporum*, *Botrytis cinerea*, *Rhizoctonia solani*, *Fusarium solani*, and *Candida albicans* at concentrations of 10–20 μM. However, it showed no inhibitory effect against *Staphylococcus aureus* or *Escherichia coli* even at 200 μM. Moreover, research by (Sener et al. 2007) confirmed that the seed oil of *Cucurbita pepo* L., cultivated in Turkey, possesses notable antibacterial activity against *Klebsiella pneumoniae* and *Acinetobacter baumannii* at 16 μg/mL, strong antifungal activity against *Candida albicans* at 8 μg/mL, and moderate antiviral activity against Parainfluenza virus type‐3 within the concentration range of 8–16 μg/mL.

### 4.4 Anthelmintic Activity

Botanical evidence and a wide array of scientific studies confirm that pumpkin seeds have long been used in traditional medicine for the treatment of prostate disorders, urinary tract ailments, and parasitic infections (Wal et al. 2024) (Figure 2). (Babaei et al. 2018) determined that a minimum effective dose of *Cucurbita maxima* seeds in mice was 23 g (approximately 73 seeds). They observed that the seeds exhibited proteolytic activity and caused structural damage to the tegument, including the basal membrane, resulting in a significant reduction in parasite egg count.

In another investigation, the anthelmintic effects of both aqueous and ethanolic extracts of *C. maxima* seeds were evaluated against *Aspiculuris tetraptera* in rat intestines (Mares et al. 2024). The results showed high efficacy: 81% for the aqueous extract and 85% for the ethanolic extract indicating superior potency of the ethanolic formulation (Ayaz et al. 2015). Similarly, (Mahmoud et al. 2002) reported that oral administration of pumpkin seed extract, prepared in boiled water and given over 14 days, led to the destruction of *Heterophyes heterophyes* parasite eggs in infected puppies.

(Elhadi et al. 2013) further explored the anti‐giardial potential of methanolic and petroleum ether extracts of *C. maxima* and *C. pepo* seeds at doses of 250, 500, and 1000 ppm. Their findings showed that *C. maxima* petroleum ether extract achieved complete (100%) efficacy against *Giardia* within 72 h at 250 ppm, and within 48 h at both 500 and 1000 ppm. In comparison, *C. pepo* exhibited 83.7% efficacy at 96 h with a 500 ppm dose. The anti‐giardial activity of *C. maxima* was attributed to triterpenes such as Cucurbitacin E and Cucurbitacin L 2‐O‐β‐glucosides.

Additionally, (Marie‐Magdeleine et al. 2009) conducted an in vitro study using water, dichloromethane, and methanol extracts from *Cucurbita moschata* seeds against *Haemonchus contortus*. Among these, only the aqueous extract effectively inhibited larval development. Moreover, (Magi et al. 2005) demonstrated that when pigs infected with *Oesophagostomum* spp. larvae were treated three times a week with pumpkin seeds at a dose of 5 g/kg, the treatment achieved a 96.1% anthelmintic efficacy comparable to ivermectin, which yielded 97.5%. These results strongly suggest that pumpkin seeds are a promising natural alternative for antiparasitic therapy.

### 4.5 Cytoprotective Effect

Certain chemical compounds are capable of protecting cells against metabolic damage induced by toxic agents, a phenomenon referred to as cytoprotection (Daoud et al. 2026). Prostaglandins represent a classical example, preventing gastric ulceration primarily through enhancement of mucosal defense rather than suppression of gastric acid secretion (Figure 3). In a similar context, several in vivo studies suggest that pumpkin seed extract (PSE) may exert cytoprotective effects against a range of toxic insults (Mohamed et al. 2026). However, the available evidence is largely derived from animal models, and its direct relevance to human health remains insufficiently established.

The reported cytoprotective activities of pumpkin seed oil and extracts (Table 5) include attenuation of emamectin‐induced toxicity in mice, as evidenced by reduced DNA fragmentation, oxidative stress, and apoptosis (Abou‐Zeid et al. 2018). PSE has also been shown to ameliorate cyclophosphamide‐induced reproductive toxicity in male rats (Samy, Abd‐Elraouf, El‐Mashad, et al. 2024) and to exhibit hepatoprotective effects against acetaminophen‐induced liver injury (Nkosi et al. 2006). Additionally, reductions in lipid peroxidation and increases in antioxidant enzyme activity, such as superoxide dismutase, have been observed in carbon tetrachloride (CCl_4_)‐induced hepatic damage models (El‐mehi 2023). While these findings are promising, they are based on controlled experimental conditions that may not fully replicate complex physiological systems in humans.

**TABLE 5:** Cytoprotective activities of pumpkin seed oil and extract.

Model	Dosage	Significance	References
In vivo emamectin‐induced oxidative stress in mice	4 mL/kg pumpkin seed oil (PSO).	Exhibited protective effects against cellular damage, including DNA fragmentation, oxidative stress, apoptosis, and alterations in gene expression.	(Abou‐Zeid et al. 2018)
In vivo bisphenol A‐induced toxicity in mice	1 mL/kg BW of PSO per day for 28 days.	Co‐administration of pumpkin seed oil (PSO) reduced DNA fragmentation and attenuated histopathological changes in testicular and hepatic tissues.	(Fawzy et al. 2018)
In vivo cyclophosphamide‐induced reproductive toxicity in adult male rats.	300 mg/kg BW of PSE for 6 weeks.	Increased total antioxidant capacity and reduced toxicity.	(Samy, Abd‐Elraouf, Almashed, et al. 2024)
In vivo acetoaminophen‐induced liver injury in Sprague–Dawley rats.	1 mL/kg BW of the pumpkin seed protein isolate	Showed significant protective activity against hepatic injury	(Hussain, Kausar, Sehar, et al. 2022a, 2022b; Hussain, Kausar, Murtaza, et al. 2022)
In vivo CCl_4_‐induced liver damage in low‐protein‐fed 52 rats was studied.	1 mL/kg BW of the pumpkin seed protein isolate.	CCl_4_‐induced liver damage was mitigated by reversing lipid peroxidation in hepatic homogenates and enhancing antioxidant defenses, including superoxide dismutase, catalase, and glutathione peroxidase, which collectively contribute to the hepatoprotective effects.	(Abdelmonsef et al. 2026)
In vivo hepatic macrovesicular steatosis and inflammation induced in high‐fat diet fed to 20‐Wistar rat.	50 g/kg BW of PSO for 422 days.	Effectively attenuated fibrosis and portal‐region cellular infiltration in the liver	(Zhao et al. 2017)

Furthermore, some studies report that PSE can reduce liver steatosis and fibrosis (Lateef et al. 2024), and may inhibit the metabolism of 1,2‐dimethylhydrazine, suggesting potential chemoprotective properties (Akomolafe et al. 2025). Nevertheless, the underlying mechanisms remain poorly defined, and it is unclear which specific bioactive compounds are primarily responsible for these effects. Variability in extraction methods, dosages, and experimental models further complicates the interpretation and comparability of results across studies.

Overall, although preliminary findings indicate potential cytoprotective and hepatoprotective effects of pumpkin seed extracts, the current body of evidence is limited by its reliance on preclinical studies. There is a clear need for standardized experimental designs, detailed mechanistic investigations, and well‐controlled clinical trials to validate these effects and assess their translational relevance.

## 5 Applications of Pumpkin Seed in Food Product Development

Pumpkin seeds (*Cucurbita* spp.), commonly dismissed as byproducts of pumpkin processing, have gained attention for their rich nutritional composition. They are dense in proteins (~30%), healthy unsaturated fats, fiber, and essential minerals like magnesium, zinc, iron, manganese, and phosphorus (Galenko et al. 2024). This profile makes them an excellent source of plant‐based nutrients and functional ingredients for various food applications. In addition to their nutritional benefits, including satiety enhancement, blood sugar regulation, cholesterol‐lowering effects, and antioxidative properties, pumpkin seeds have gained recognition in wellness trends (Öztürk and Turhan 2020). Such attributes have led to their incorporation in snacks, dietary supplements, and fortified foods. Pumpkin seeds are recognized as a functional food due to their pharmacological and biofunctional properties (Gomez‐Garcia et al. 2020). Recent studies have explored their use in food formulations, such as biscuits, where pumpkin seeds were shown to enhance protein content when added to casein‐based diets (Hussain, Kausar, Sehar, et al. 2022a, 2022b; Hussain, Kausar, Murtaza, et al. 2022). However, gluten‐free protein formulations face challenges due to texture issues, largely due to the absence of gluten proteins. Studies demonstrate that plant protein enrichment, particularly with pumpkin seeds, can improve the texture and protein content of gluten‐free bread (Šmídová and Rysová 2022). This has led to the production of baked goods with improved textural properties and water‐holding capacities (Skendi et al. 2021).

### 5.1 Bread and Flour Fortification

Pumpkin seed flour or protein concentrate can be incorporated into wheat flour to raise protein, lysine, and mineral content without negatively affecting dough handling or loaf quality. For example, up to 21% pumpkin seed protein isolate or flour can be used to fortify bread, improving nutrition and texture (El‐Soukkary 2000). Protein‐rich pumpkin seed concentrate enhances dough gas retention and structural integrity while elevating unsaturated fat content and digestibility (Galenko et al. 2024). Consumers accept up to 10% addition levels in bread, suggesting a viable route to healthier bakery goods (Galenko et al. 2024).

### 5.2 Cookies, Crackers, Cakes, and Gluten‐Free Baked Goods

Pumpkin seed flour enhances sensory attributes and nutritional value in various baked products. Adding 5%–10% pumpkin seed powder maintains crust color and flavor of sponge cake, without detrimental effects (Pandey et al. 2024). Replacing 20%–35% chickpea flour with pumpkin seed cake flour boosts protein, fiber, phenolics, antioxidant activity, and lowers glycemic index of crackers all without weakening taste (Pandey et al. 2024). A study was conducted to evaluate the potential application of pumpkin seed powder in sponge cake production (Pandey et al. 2024). Sponge cakes were prepared by incorporating 5% and 10% pumpkin seed powder, and their physical and sensory properties were compared with those of a control sample. The results indicated that the crust color of the pumpkin seed‐enriched cakes was comparable to that of the control, suggesting no significant alteration in visual appeal. Moreover, the pumpkin seed‐incorporated sponge cakes exhibited favorable qualitative characteristics, confirming their suitability for product development (Goranova et al. 2019).

In a separate formulation study, three types of crackers were developed: a control made entirely with chickpea flour, and two variations in which 20% and 35% of the chickpea flour were replaced with cold‐pressed pumpkin seed cake flour (Pandey et al. 2024). The proximate analysis of the crackers revealed that increasing levels of pumpkin seed cake flour enhanced the protein, fat, and ash content, while decreasing the overall carbohydrate content. These results highlight the nutritional enrichment potential of pumpkin seed derivatives in baked snack products. The proximate composition of the crackers was influenced by the stepwise substitution of chickpea flour with pumpkin seed press cake flour (Tomić et al. 2022). This replacement led to increased levels of protein, fat, and ash, while contributing to a reduction in total carbohydrate content. Given the high dietary fiber and protein content of the raw materials used, the resulting crackers could be marketed with nutritional claims such as “high in fiber” and “a source of protein.”

Furthermore, the inclusion of pumpkin seed press cake flour resulted in a progressive enhancement of total phenolic content and antioxidant activity (Tomić et al. 2022). Although all cracker samples demonstrated a moderate glycemic index, the substitution of chickpea flour with pumpkin seed press cake flour at both 20% and 35% levels significantly lowered the glycemic index (Pandey et al. 2024). Sensory evaluation confirmed that all tested cracker variants possessed acceptable organoleptic properties. Notably, the presence of pumpkin seed press cake flour not only maintained but even improved certain sensory attributes, including taste and flavor. This study highlights the potential of utilizing pumpkin seed press cake as a valuable food industry by‐product in the production of gluten‐free crackers (Tomić et al. 2024). Its application contributes to the nutritional enhancement of the final product, boosts antioxidant properties, reduces glycemic response, and supports the sustainable use of agro‐industrial residues, aligning with the principles of circular economy and functional food development (Granato et al. 2022).

(Norfezah et al. 2011) investigated the incorporation of flours derived from three fractions: peel, flesh, and seed of crown pumpkin ( *Cucurbita maxima* ) into extruded snack formulations. The mixtures were processed using a twin‐screw extruder to produce 10 types of expanded snack products. Incorporation of peel and seed flours at 10% resulted in products with expansion and density comparable to the control. However, inclusion levels exceeding 10% led to increased product hardness.

## 6 Conclusion

Pumpkin seeds are a nutritionally dense and functionally valuable food ingredient with considerable potential for human health and food innovation. Despite extensive documentation of their composition and bioactive compounds, several knowledge gaps remain, particularly regarding the bioavailability, metabolism, and clinical efficacy of their phytosterols, antioxidants, cucurbitacins, lignans, and tryptophan in humans. Most evidence supporting health benefits is derived from in vitro or animal studies, highlighting a limitation in translating these effects to human populations. Future research should focus on well‐designed clinical trials, standardized extraction and processing methods, and mechanistic studies to clarify their physiological impacts. Additionally, challenges in food formulation, stability, and sensory acceptance need to be addressed to maximize the functional integration of pumpkin seeds in diverse food systems. Addressing these gaps will strengthen the scientific foundation for their use as a functional food ingredient and support the development of sustainable, health‐promoting dietary solutions.

## Author Contributions

**Messenbet Geremew Kassa:** conceptualization, writing – original draft, writing – review and editing, methodology. **Desye Alemu Teferi:** writing – review and editing.

## Funding

There is no funding for this project.

## Conflicts of Interest

The authors declare no conflicts of interest.

## Data Availability

The data that support this review is available from the corresponding author upon reasonable request.
